# Laryngeal metastasis from a thumb melanoma after a 5-year disease-free interval: A case of acute airway presentation and review of the literature

**DOI:** 10.1016/j.radcr.2026.01.093

**Published:** 2026-03-05

**Authors:** Achraf Amine Sbai, Issam Berrajaa, Mohamed Bouallou, Drissia Benfadil, Azeddine Lachkar, Fahd El Ayoubi El Idrissi

**Affiliations:** a Faculty of Medicine and Pharmacy, Mohammed Ist University, Oujda, Morocco; b Department of Otorhinolaryngology, Mohammed VI University Hospital, Oujda, Morocco

**Keywords:** Laryngeal metastasis, Metastatic melanoma, Case report, Immunotherapy, Laryngectomy

## Abstract

Metastatic malignant melanoma to the larynx is an exceptionally rare clinical entity. We report the case of a 55-year-old woman with a history of thumb melanoma treated 5 years prior, who presented with laryngeal dyspnea. Imaging revealed a large, non-enhancing laryngeal mass infiltrating all 3 levels, with thyroid cartilage lysis and an ipsilateral metastatic cervical lymph node. Following emergency tracheotomy, histopathology confirmed metastatic melanoma (HMB-45+, Melan-A+). The patient underwent total laryngectomy with bilateral neck dissection, followed by adjuvant nivolumab immunotherapy. At the 1-year follow-up, she shows no evidence of recurrence with good stoma function and manageable immunotherapy-related fatigue. This case highlights the diagnostic challenges, the potential for aggressive curative treatment in oligometastatic disease, and underscores the need for lifelong surveillance in melanoma patients.

## Introduction

Mucosal melanoma is a rare entity, accounting for a small proportion of all melanomas, with approximately 55% located in the head and neck region [1,2]. This rarity can be explained, in part, by the neuroectodermal origin of melanocytes, which contrasts with the endodermal origin of the mucosae where they are exceptionally found [3].

Metastases to the mucosa from a primary cutaneous melanoma are also uncommon. Approximately 0.6% to 9.3% of patients with cutaneous melanoma develop metastases in the mucosa of the upper aerodigestive tract, of which only about 12% involve the larynx [4]. This scarcity explains the absence of clear therapeutic guidelines in the literature, with management often being individualized and guided by clinical experience and the specific characteristics of each case.

The diagnosis of mucosal melanoma relies on histopathological and immunohistochemical criteria, including the demonstration of intracellular melanin, tyrosinase activity, and the presence of premelanosomes. Immunohistochemistry generally confirms the diagnosis through positivity for markers such as HMB-45, S-100, MART-1/Melan A, vimentin, and NKI/C3 [5,6]. Certain biological markers like LDH, protein S-100B, or 5-S-cysteinyldopa have been well-correlated with disease progression and survival [[7], [8], [9]], though their utility in clinical practice remains debated.

Mucosal melanomas are characterized by marked aggressiveness, with a tendency for local invasion, satellite formation, angiolymphatic extension, and frequent regional or distant dissemination [[10], [11], [12]]. Despite this, the standard treatment remains primarily surgical, albeit with high rates of local recurrence and metastasis.

In this context, we report the case of a 55-year-old female patient presenting with a laryngeal metastasis from a malignant melanoma of the thumb, operated on 5 years earlier. Through this case, we illustrate not only the rarity of this location but also the diagnostic and therapeutic challenges posed by the lack of a management consensus in the literature.

## Case description

A 55-year-old woman with type 2 diabetes presented with progressively worsening laryngeal dyspnea, dysphonia, and stridor. Her history included a non-ulcerated malignant melanoma of the left thumb (Breslow thickness >1 mm) treated 5 years prior by surgical amputation (function-preserving) with clear margins (≥1 cm). The initial histopathology placed her in an intermediate-to-high-risk category. She received no adjuvant therapy and was on regular surveillance with annual dermatological exams and regional ultrasounds, all unremarkable until this acute presentation.

On admission, she was in moderate respiratory distress with desaturation to 75% on room air, supraclavicular retraction, and thoraco-abdominal breathing. Examination revealed a firm, fixed left jugulocarotid lymph node. Nasofibroscopy showed a purplish, exophytic mass occupying the interarytenoid region and extending to the ventricular bands and anterior commissure.

**Fig. 1:**
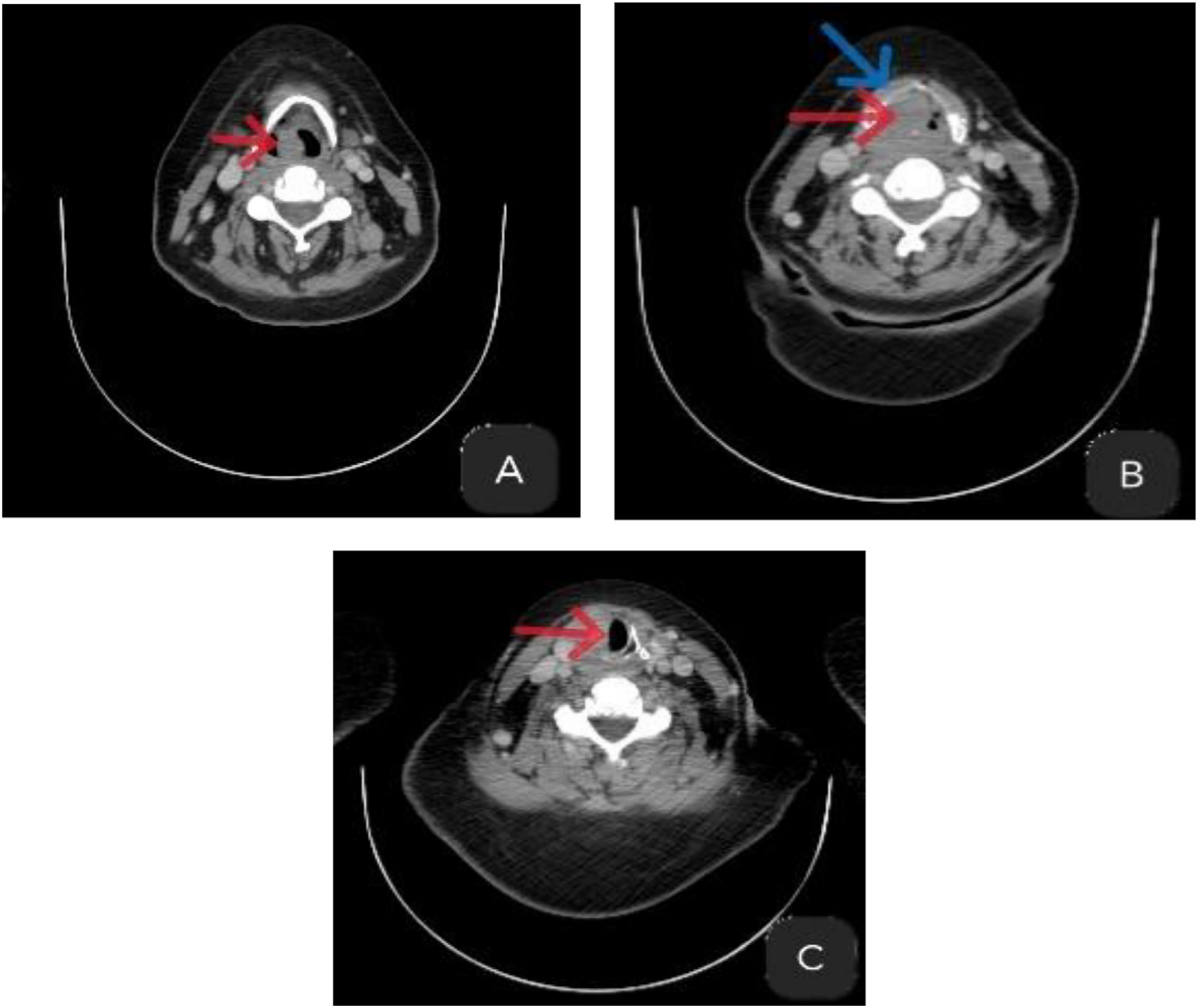
Axial contrast-enhanced computed tomography (CT) images of the neck (soft-tissue window). The red arrow indicates the primary tumor mass; the blue arrow indicates cartilage lysis. (A) Supraglottic level: large, exophytic mass (red arrow) with associated destruction of the thyroid cartilage (blue arrow). (B) Glottic level. (C) Subglottic level.

An urgent contrast-enhanced cervical CT scan (portal venous phase, 1.0 mm reconstructions) revealed a large, exophytic, and transglottic laryngeal mass. The tumor arose from the supraglottis with extensive inferior involvement, causing severe narrowing of the laryngeal airway at all levels. It measured approximately 3.2 cm in craniocaudal, 2.2 cm in transverse, and 2.4 cm in anteroposterior dimensions. It showed heterogeneous post-contrast enhancement and osteolytic destruction of the thyroid and arytenoid cartilages, with involvement of the superior rim of the cricoid cartilage. A left level II lymph node, measuring 27 mm in its short axis and exhibiting necrotic features, was highly suggestive of metastasis (Fig. 1). There was no radiologic evidence of invasion into the pre-epiglottic space, paraglottic spaces, hypopharynx, or trachea.

**Fig. 2:**
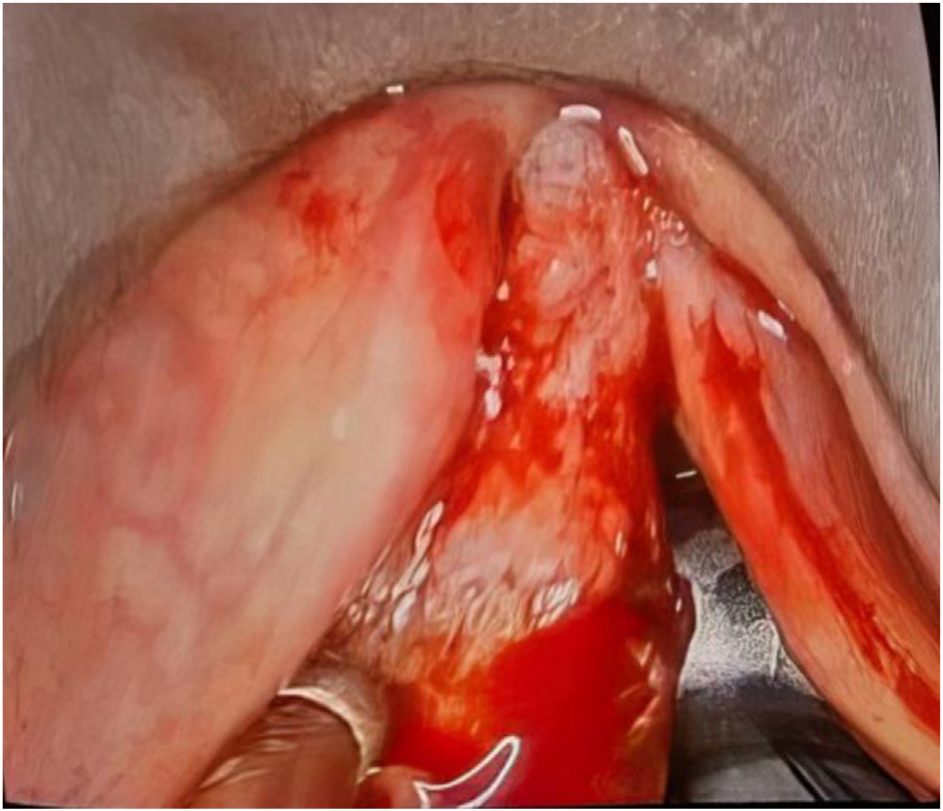
Intraoperative view during direct laryngoscopy showing laryngeal melanoma.

Due to the imminent risk of respiratory distress, an emergency tracheotomy was performed, and direct laryngoscopy with biopsy of the laryngeal mass was carried out (Fig. 2).

**Fig. 3:**
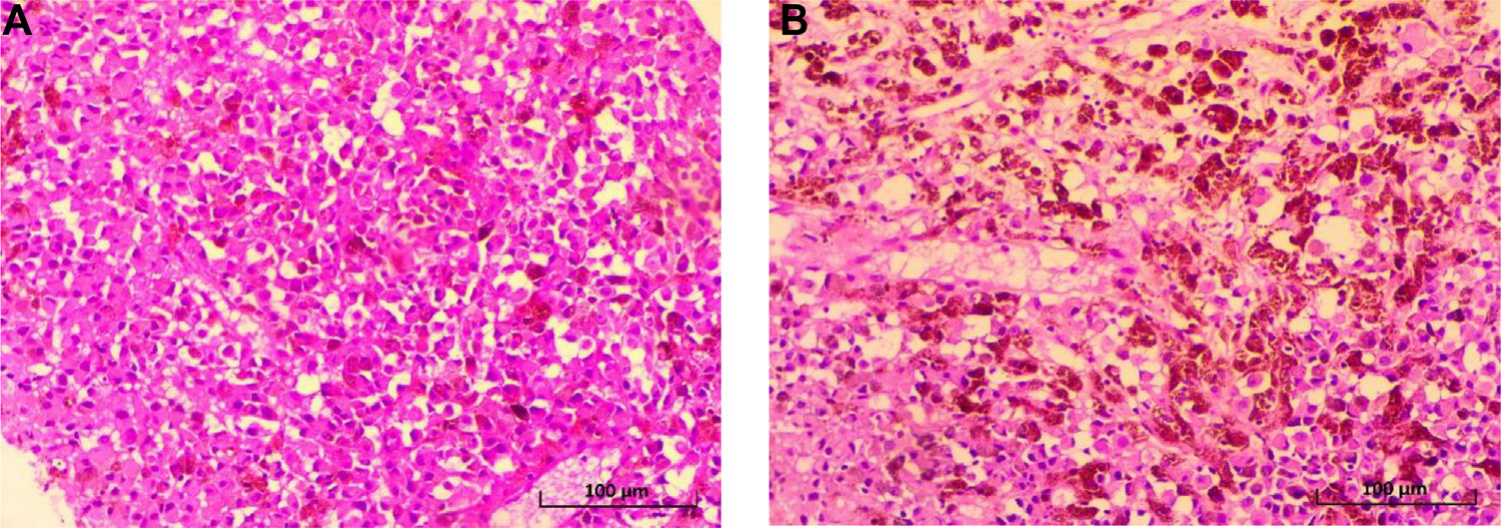
(A) Histological section of the malignant melanoma showing a dense proliferation of atypical melanocytes (Hematoxylin and Eosin stain, x400). (B) Histological image showing cellular details of the melanoma. The pigmented appearance of the tumor cells should be noted (Hematoxylin and Eosin stain, x400).

Histopathological analysis of the biopsy specimen revealed a malignant tumor proliferation with both rounded and spindle-shaped cells, dissociating the mucous glands without continuity with the surface epithelium. This proliferation consisted of cells exhibiting significant nuclear pleomorphism and a high mitotic index. Numerous tumor cells contained cytoplasmic brown-blackish melanin pigment. Critically, there was no evidence of an in situ component, junctional activity, or atypical melanocytic hyperplasia in the overlying or adjacent mucosal epithelium, effectively ruling out a primary mucosal melanoma. The histological features were therefore consistent with a metastatic deposit (Figs. 3A and B).

**Fig. 4:**
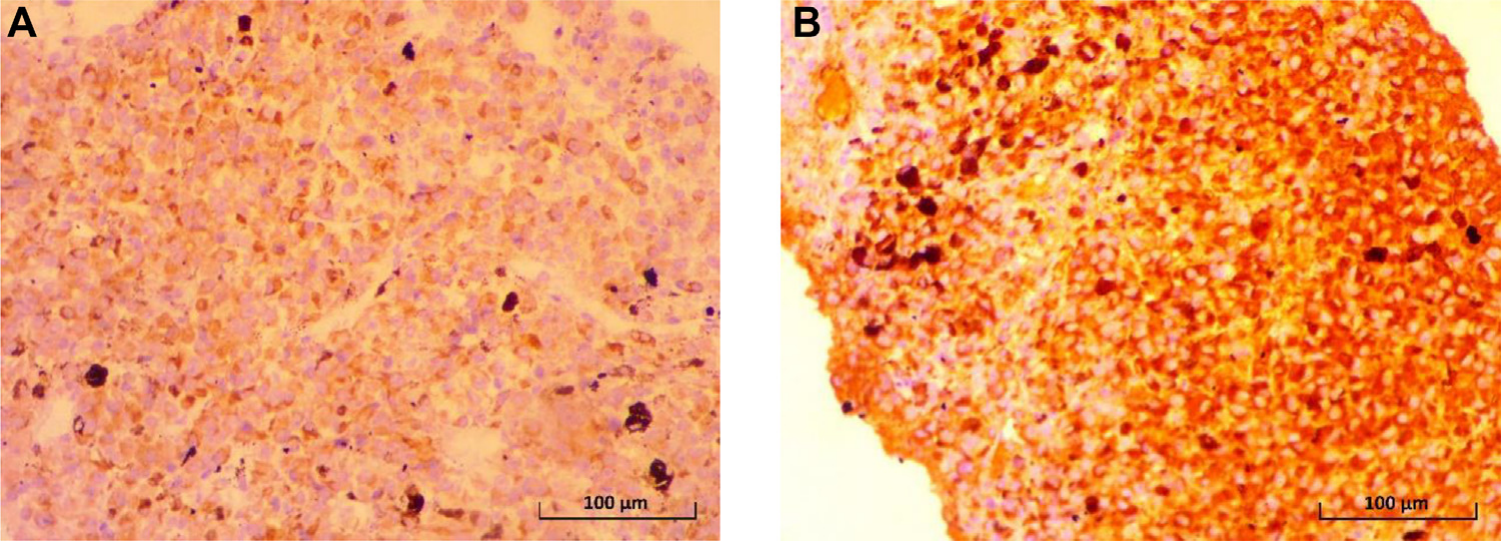
(A) Immunohistochemical image showing positivity for Anti-Melan A antibody (IHC, x400). (B) Immunohistochemical image showing positivity for Anti-HMB-45 antibody (IHC, x400).

To confirm the diagnosis, a comprehensive immunohistochemical study was performed. The tumor cells showed strong, diffuse, and co-expression of the specific melanocytic markers Melan-A (MART-1) and HMB-45. They were also diffusely positive for SOX10 and showed strong nuclear positivity for MITF, further consolidating the diagnosis of melanoma. This complete immunoprofile (Melan-A+, HMB-45+, SOX10+, MITF+), characteristic of melanocytic differentiation, confirmed the diagnosis of metastatic malignant melanoma (Figs. 4A and B).

A full systemic staging workup was performed using contrast-enhanced computed tomography (CT) of the chest, abdomen, and pelvis—the standard imaging for initial staging of metastatic melanoma in our institution. This comprehensive evaluation confirmed the absence of distant metastases beyond the known laryngeal and cervical sites.

The complete case was discussed at a multidisciplinary tumor board (MTB). The decision to proceed with a radical surgical approach via total laryngectomy was based on the following rationale: The presence of acute airway obstruction necessitating an urgent procedure with both curative and salvage intent; The transglottic extent of the tumor, involving all 3 laryngeal levels with cartilage destruction, which precluded the possibility of partial or larynx-preserving surgery with clear margins; The known radioresistant nature of melanoma, making primary or neoadjuvant radiotherapy poorly effective for durable local control; The absence of distant metastases (oligometastatic disease), which presented an opportunity for definitive local control via complete excision. A systemic therapy-first approach (eg, immunotherapy) would have carried an unacceptable risk of local tumor progression during the therapeutic latency period, potentially leading to an inoperable situation or a loss of curative opportunity.

**Fig. 5:**
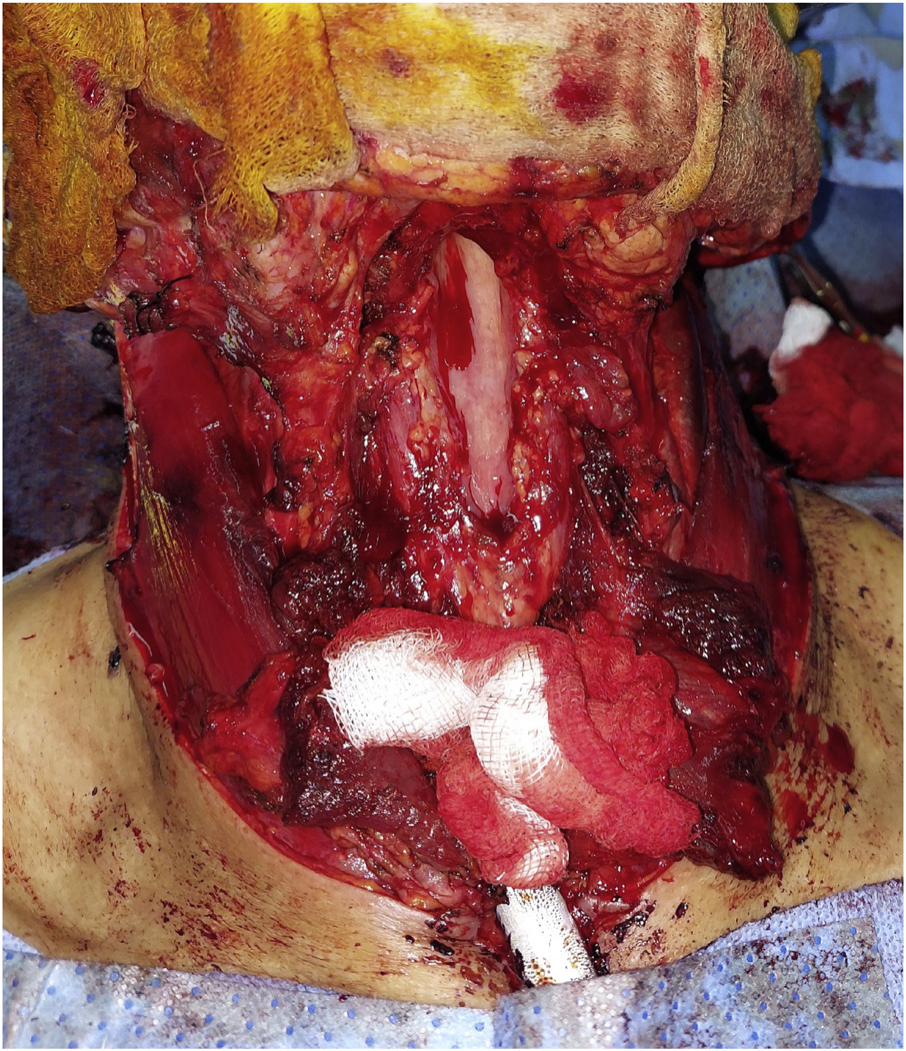
Intraoperative view after total laryngectomy.

**Fig. 6:**
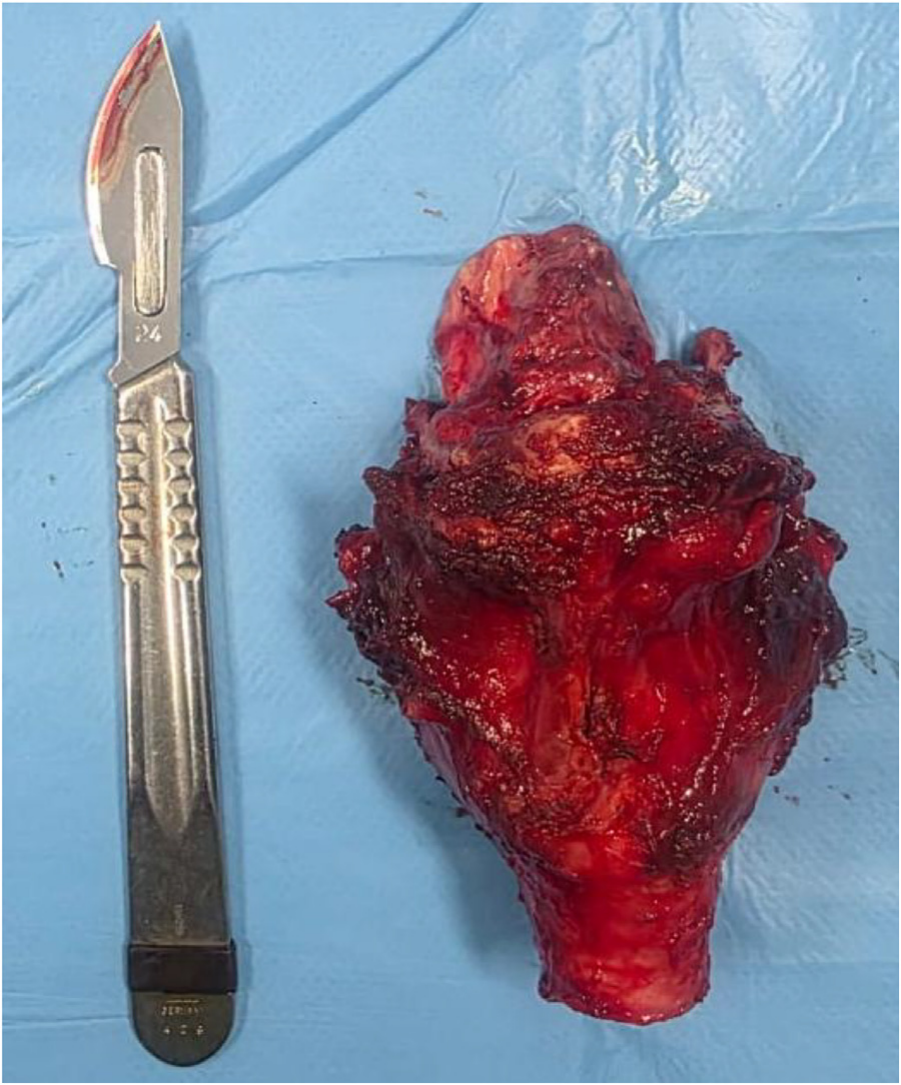
Surgical specimen of total laryngectomy.

**Fig. 7:**
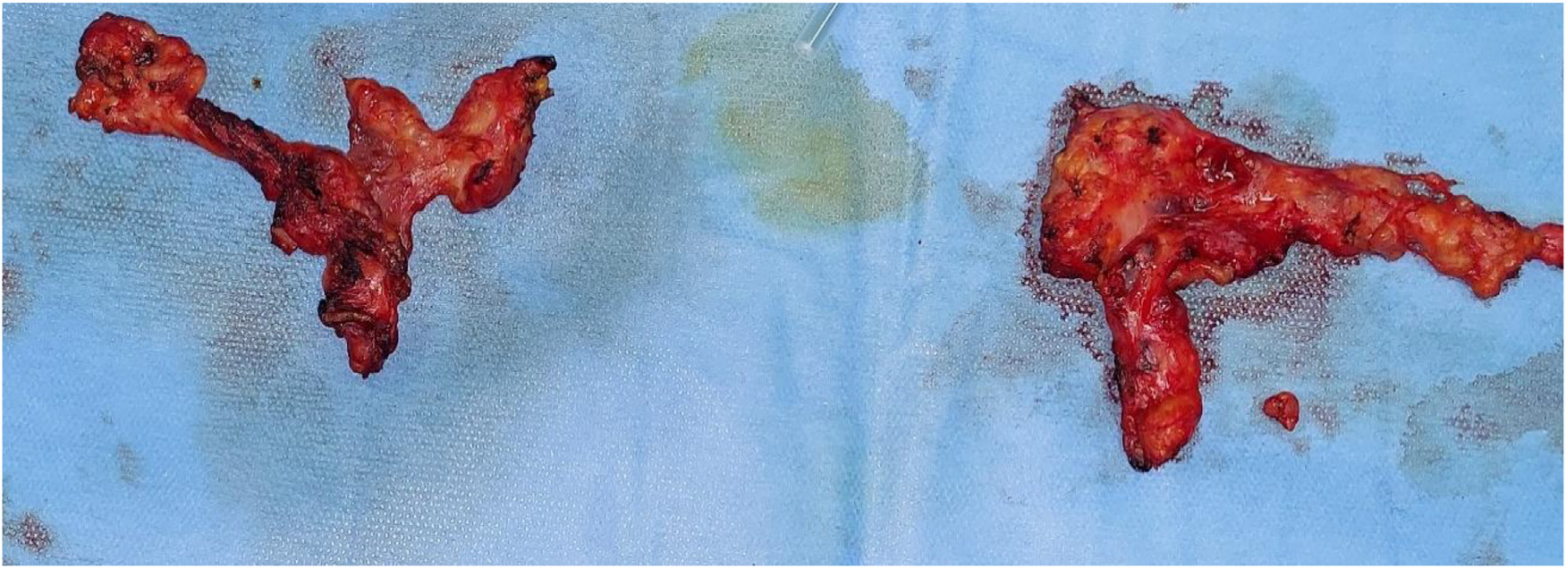
Surgical specimens from lymph node dissection.

The patient therefore underwent a total laryngectomy (Figs. 5 and 6) with bilateral cervical lymph node dissection (levels II to IV) (Fig. 7). The definitive histopathological analysis confirmed metastatic melanoma with clear resection margins (R0). The lymphadenectomy specimen yielded 32 lymph nodes, of which 2 from the left level II were positive for metastatic melanoma (2/32, pN1b stage). No extracapsular extension was identified. The limited nodal burden (2 positive nodes out of 32 examined) supported the preoperative assessment of oligometastatic disease and the decision for aggressive local therapy followed by adjuvant immunotherapy systemic treatment.

A complementary immunohistochemical study was performed on the tumor specimen to assess PD-L1 protein expression. This showed positive expression in less than 1% of tumor cells, defining a PD-L1 negative status.

Based on clinical trials demonstrating a significant improvement in recurrence-free survival in stage III melanoma, independently of PD-L1 status, the therapeutic decision was to administer anti-PD1 immunotherapy. These clinical trials are detailed further in the discussion.

The patient was referred to the oncology department for the initiation of adjuvant treatment with nivolumab, planned for 1 year.

## Postoperative Course and Follow-up

The patient’s postoperative recovery was uneventful. The tracheostomy cannula was removed on the seventh postoperative day, and the stoma healed well without complications. Adjuvant immunotherapy with nivolumab (480 mg every 4 weeks) was initiated 8 weeks after surgery.

*Treatment tolerance:* The treatment was well-tolerated. The patient experienced grade 1 fatigue (according to CTCAE v5.0 criteria), which was managed conservatively without dose modification or treatment interruption. No significant immune-related adverse events (eg, colitis, pneumonitis, hepatitis, or endocrinopathies) were observed during the treatment period.

*Oncologic Surveillance:* Regular follow-up was conducted with clinical examination and imaging.

• *At 3 months:* A neck ultrasound showed no evidence of local recurrence or pathological lymphadenopathy.
• *At 6 months:* A restaging whole-body CT scan confirmed the absence of local, regional, or distant metastatic disease.
• *At the most recent follow-up (12 months post-surgery):* The patient remains clinically and radiologically disease-free. She continues to receive nivolumab with good functional status and stable, manageable side effects.

## Discussion

Malignant melanoma is an aggressive tumor, with mucosal localizations being rare and accounting for approximately 1% of all melanoma cases [1]. Among these, the head and neck region constitutes the site of more than 50% of mucosal melanomas, primarily located in the nasal cavities, paranasal sinuses, oral cavity, and more rarely, the larynx [13,14].

The case we report illustrates an unusual progression of a cutaneous melanoma of the thumb, which occurred 5 years earlier, resulting in an isolated laryngeal metastasis associated with left cervical lymph node involvement. This disease course is extremely rare. An in-depth search of the PubMed database, conducted by Santos RS et al. in the journal Life (2023), found only a few published cases of laryngeal metastases from cutaneous melanomas, confirming the exceptional rarity of this presentation [6].

The 5-year interval between the primary digital tumor and the laryngeal metastasis warrants particular attention. It highlights the capacity of melanoma to cause late recurrences, a well-documented phenomenon whose mechanisms—often involving tumor dormancy—remain incompletely understood. The occurrence of a regional (lymph node) metastasis synchronous with the laryngeal metastasis, yet in the absence of any visceral dissemination, is also noteworthy. This dissemination pattern, bypassing the classic hematogenous spread in favor of an aggressive locoregional progression, is atypical and might suggest a particular tropism of the melanocytic cells for the laryngeal mucosal microenvironment or a unique tumor biology.

Clinically, laryngeal metastases typically present with laryngeal dyspnea, dysphonia, stridor, or acute airway obstruction, as observed in our patient. Lanson et al. emphasize that the supraglottic region is the most frequently involved site in cases of laryngeal metastatic deposits [15,16]. The tumor in our case exhibited an extensive character from the outset, invading all 3 laryngeal levels. This anatomical particularity explains the rapid progression and the necessity for an emergency tracheotomy.

Diagnostically, histopathology coupled with immunohistochemistry remains the gold standard. Positivity for S-100 protein, HMB-45, and Melan-A, as observed in our case, confirms the melanocytic origin of the tumor. Distinguishing between primary and metastatic forms relies notably on the presence or absence of junctional activity and pre-existing mucosal architecture, as described in the article by Silva Leandro Santos et al. [17].

Regarding treatment, curative therapy relies on complete surgical resection with negative margins when possible, as indicated by the Australian and American guidelines referenced by Laurence Pincet et al. [18]. In our case, an aggressive surgical approach was deemed feasible due to the absence of distant metastases. The patient therefore underwent total laryngectomy with bilateral cervical lymph node dissection, followed by adjuvant immunotherapy, in accordance with the current paradigm for managing high-risk of recurrence melanoma.

The role of radiotherapy in melanoma remains debated. Although hypofractionated protocols are proposed due to melanoma's radioresistance, clinical outcomes are inconsistent. In cases of positive margins or surgical contraindications, palliative radiotherapy at doses of 60 to 66 Gy may be considered [16].

Concerning immunotherapy, checkpoint inhibitors such as nivolumab, pembrolizumab (anti-PD1), and ipilimumab (anti-CTLA4) have shown notable efficacy in metastatic cutaneous melanoma. However, results appear more modest for mucosal forms, as indicated by recent data reported by Pincet et al. [18]. Based on pivotal clinical trials demonstrating a significant improvement in recurrence-free survival in resected stage III melanoma, independently of PD-L1 status, the therapeutic decision was to administer anti-PD1 immunotherapy. The CheckMate 238 trial established that adjuvant nivolumab, compared to ipilimumab, significantly reduced the risk of recurrence with a more favorable safety profile in patients after complete resection of stage IIIB/C or IV melanoma [19]. Similarly, the KEYNOTE-054 trial showed that adjuvant pembrolizumab substantially improved recurrence-free survival compared to placebo in patients with stage III melanoma with lymph node involvement [20]. This evidence, which applies to cutaneous melanoma, constitutes the best available standard of care to guide the management of a rare mucosal metastasis in a similar context of high-risk resected disease. In our case, adjuvant anti-PD1 immunotherapy was indicated to reduce the risk of recurrence following complete surgical excision.

The prognosis for laryngeal metastases from melanoma remains poor. The median survival reported in the literature ranges between 2 and 8 months after diagnosis of mucosal metastasis [21]. Our case is distinguished by the absence of systemic dissemination at the time of the laryngeal metastasis diagnosis, a more favorable situation that permitted aggressive surgical management. Furthermore, Life 2023 mentions that only 5% of patients with initially localized melanoma develop late metastases, but these forms are particularly aggressive with an estimated 10-year survival rate between 4.5% and 8% [22,23].

Finally, although some studies suggest a more unfavorable prognosis when the primary tumor is located in the head and neck, these results vary across series. In our case, the initial digital site further complicates the prognostic interpretation.

## Conclusion

The case we report illustrates the diagnostic and therapeutic challenge posed by a late laryngeal metastasis of cutaneous melanoma. It underscores the imperative need for prolonged clinical surveillance and high vigilance regarding any ENT symptom, even several years after management of the primary tumor. The rarity of this entity should not lead to a systematic palliative approach, as demonstrated by our experience where an aggressive multidisciplinary management, combining complete surgical excision and adjuvant immunotherapy, was successfully implemented. The publication of such cases is crucial to enrich the still limited data and to optimize management strategies for these rare metastatic forms.

## Ethical approval

According to local guidelines, ethical approval was not required for this case report, as it does not involve experimental research on human subjects. However, written informed consent was obtained from the patient for publication of the case

## Data availability statement

All data supporting the findings of this study are available from the corresponding author upon reasonable request. No publicly archived datasets were generated or analyzed during the current study.

## Patient consent

Written informed consent was obtained from the patient for publication and any accompanying images. A copy of the written consent is available for review by the Editor-in-Chief of this journal on request.
